# Use of FabV-Triclosan Plasmid Selection System for Efficient Expression and Production of Recombinant Proteins in *Escherichia coli*

**DOI:** 10.1371/journal.pone.0144189

**Published:** 2015-12-07

**Authors:** Syed A. Ali, Yik Wei Chew, Tasyriq Che Omar, Nizuwan Azman

**Affiliations:** 1 Oncological and Radiological Sciences, Advanced Medical and Dental Institute, Universiti Sains Malaysia, Bertam, 13200, Kepala Batas, Pulau Pinang, Malaysia; 2 Division of Research Publications, and Innovation, Advanced Medical and Dental Institute, Universiti Sains Malaysia, Bertam, 13200, Kepala Batas, Pulau Pinang, Malaysia; Universidad de Palermo, UNITED STATES

## Abstract

Maintenance of recombinant plasmid vectors in host bacteria relies on the presence of selection antibiotics in the growth media to suppress plasmid -free segregants. However, presence of antibiotic resistance genes and antibiotics themselves is not acceptable in several applications of biotechnology. Previously, we have shown that FabV-Triclosan selection system can be used to select high and medium copy number plasmid vectors in *E*. *coli*. Here, we have extended our previous work and demonstrated that expression vectors containing FabV can be used efficiently to express heterologous recombinant proteins in similar or better amounts in *E*. *coli* host when compared with expression vectors containing β-lactamase. Use of small amount of non-antibiotic Triclosan as selection agent in growth medium, enhanced plasmid stability, applicability in various culture media, and compatibility with other selection systems for multiple plasmid maintenance are noteworthy features of FabV-Triclosan selection system.

## Introduction

Plasmid vectors frequently employ antibiotic resistance genes as selection markers; consequently, growth media are regularly supplemented with antibiotics. [1]. Nevertheless, use of antibiotics is unwelcomed in certain biotechnology applications such as gene therapy and production of therapeutic recombinant proteins [2]. Besides, for large-scale fermentation, plasmid vector selection using antibiotics can prove expensive [3]. Disadvantages associated with antibiotic -based selection have encouraged development of antibiotic marker -free selection approaches. Several such systems have been reported [4, 5, 6, 7, 8, 9, 10, 11] but not adopted commonly by research laboratories for numerous reasons that, among other, include use of specialized strains and/or reagents.

Triclosan, a polychloro phenoxy phenol, is a Food and Drug Administration (FDA) -approved non-antibiotic biocide agent present in many consumer products that include toothpastes, mouthwashes, soaps, shampoos, deodorants etc. [12, 13]. Triclosan inhibits bacteria by binding to bacterial enoyl-acyl carrier protein reductase enzyme (ENR), which is encoded by *fab*I gene in *Escherichia coli* (*E*. *coli*). Triclosan inhibits ENR through binding at the ACP-enoyl substrate site, forming a stable Triclosan/NAD^+^/ENR ternary complex. When overexpressed, ENR confers resistance towards Triclosan -mediated growth inhibition of *E*. *coli* [14]. Therefore overexpression of *fab*I is suggested as another plasmid selection marker in place of unwanted antibiotic markers such as β-lactamase [15, 16, 17].

We found out that overexpression of *fab*I can be used to select high copy number (~500–700 copies per bacteria) pUC -based plasmids but not medium copy number (~15–20 copies per bacteria) pBR322 -based plasmids. It appeared that *fab*I, when expressed under its own or weak P3 promoter and from pBR322 -based vectors, did not produce sufficient ENR to confer resistance towards Triclosan [18]. Since overexpression of a cloned gene or its protein product may exert deleterious effects on host bacteria, medium copy number plasmid vectors are desirable for heterologous gene expression and protein production [19]. We found that overexpression of *fab*V, a functional homologue of *fab*I in *Vibrio cholera*, under P3 promoter and from pBR322 -based vectors conferred resistance towards Triclosan and can be used to select pBR322 -based vectors in *E*. *coli* [18].

Here, we have extended our previous work [18] and demonstrated that *fab*V-Triclosan selectable medium copy number plasmid vectors can be used efficiently to express heterologous recombinant proteins in similar or better amounts in *E*. *coli* when compared with traditional *bla* (β-lactamase)-Ampicillin selectable vectors.

## Material and Methods

### Bacterial strains and culture conditions

The *E*. *coli* strain DH5α (NEB, #C2987) was used for cloning and plasmid propagation purposes whereas BL21(DE3) (NEB, #C2527) was used for protein expression. Bacterial strains were grown aerobically in LB (Miller) broth, or on LB (Miller) agar at different temperatures, and in the presence or absence of Ampicillin (100 μg mL^-1^) or Triclosan (1 μM). Bacterial strains were stored in glycerol (50%) -supplemented LB broth at -80°C.

### General molecular biology techniques

PCR amplifications of DNA fragments intended for cloning purposes was carried out using Q5 High-Fidelity DNA Polymerase (NEB, #M0491), whereas Taq DNA Polymerase (NEB, #M0273) was used for routine colony PCR. PCR amplified or restricted DNA fragments were purified using NucleoSpin Gel and PCR Clean-up kit (Macherey-Nagel GmbH & Co, #740609). Plasmid DNA was purified using Wizard^®^ Plus SV Minipreps DNA Purification System (Promega, #A1465). Vector and insert were mixed in 1:3 molar ratios (unless otherwise specified) and ligated in presence of T4 DNA ligase (NEB, #M0202) at 4°C for 18 h. For colony PCR, at least 10 randomly selected bacterial colonies were PCR amplified using a vector -specific and an insert -specific primer (Table 1). Plasmid DNA was isolated from positive transformants and verified using DNA restriction and sequence analyses.

**Table 1:** Plasmid vectors used in this study.

Vector	Essential features
**pSA-HP24-Bla (6.595 kb)**	
	pBR322 ori.
	Constitutively expresses *bla* (Amp^R^) under P3 promoter.
	Expresses HIV-1 *p24* under IPTG–inducible T7 promoter.
	Referred to as pSA-HP24-6His and described in [20].
**pSA-HP24-FabV (6.950 kb)**	
	pBR322 ori.
	Constitutively expresses *fabV* (Trc^R^) under P3 promoter.
	Expresses HIV-1 *p24* under IPTG–inducible T7 promoter.
	Described in [18]
**pSA-HNef-Bla (6.517 kb)**	
	pBR322 ori.
	Constitutively expresses *bla* (Amp^R^) under P3 promoter.
	Expresses HIV-1 *nef* under IPTG–inducible T7 promoter
	Referred to as pSA-HNef-6His and described in [21]
**pSA-HNef-FabV (6.872 kb)**	
	pBR322 ori.
	Constitutively expresses *fabV* (Trc^R^) under P3 promoter.
	Expresses HIV-1 *nef* under IPTG–inducible T7 promoter.
	Engineered in this study.
**pSA-HVif-Bla (6.5 kb)**	
	pBR322 ori.
	Constitutively expresses *bla* (Amp^R^) under P3 promoter.
	Expresses HIV-1 *vif* under IPTG–inducible T7 promoter
	Referred to as pSA-HVif-6His and described in [21].
**pSA-HVif-FabV (6.833 kb)**	
	pBR322 ori.
	Constitutively expresses *fabV* (Trc^R^) under P3 promoter.
	Expresses HIV-1 *vif* under IPTG–inducible T7 promoter.
	Engineered in this study

### Plasmid construction

A total of six plasmid vectors were used in this study (Table 1). Construction of pSA-HP24-Bla [20], pSA-HP24-FabV [18], pSA-HNef-Bla [21], and pSA-HVif-Bla [21] is described previously. Two new plasmids, pSA-HNef-FabV and pSA-HVif-FabV were constructed in this study. The pSA-HNef-FabV and pSA-HVif-FabV were derived from pSA-HNef-Bla and pSA-HVif-Bla respectively. The pSA-HNef-Bla and pSA-HVif-Bla were whole plasmid PCR amplified by using pMXB10-out-*bla*-*Cla*I-R and pMXB10-out-*bla*-*Sma*I-F primers (Table 2). The *fab*V gene was PCR amplified from genomic DNA of *Vibrio cholerae* O1 El Tor by using FabV-*Cla*I-F and FabV-*Sma*I-R primers (Table 2). Vector and insert amplicons were purified and sequentially restricted with *Cla*I and *Sma*I endonucleases. Restricted vector and insert were purified, ligated, and transformed into chemically competent DH5α *E*. *coli*. Transformants were selected on LB agar plates containing 1 μM Triclosan after 18 h of incubation at 30°C. Clones were verified by colony PCR using vector -specific PMXB10-up101-F and insert -specific FabV-*Sma*I-R primers (Table 2).

**Table 2:** Oligonucleotides used to construct vectors used in this study.

Primer	Sequence (5’ → 3’)
pMXB10-out-*bla*-*Cla*I-R	GGACATCGATACTCTTCCTTTTTCAATATTATTG
pMXB10-out-*bla*-*Sma*I-F	GTAACCCGGGCTGTCAGACCAAGTTTACTCA
FabV-*Cla*I-F	GAGTATCGATGATCATCAAACCTAAAATTCGT
FabV-*Sma*I-R	ACAGCCCGGGTTACTCGATATCAATCACATCGAA
PMXB10-up101-F	GATCCCGCGAAATTAATACG

### Expression of proteins

Sequencing -confirmed expression vectors were transformed into BL21(DE3) *E*. *coli*. Those transformed with pSA-HP24/HNef/HVif-Bla vectors were selected on LB agar plates containing 269 μM (100 μg mL^-1^) Ampicillin whereas bacteria transformed with pSA-HP24/HNef/HVif-FabV vectors were selected on LB agar plates containing 1 μM Triclosan. For expression experiments, a single colony from a freshly streaked (18–22 h) LB agar selection plate was inoculated into 10 ml of culture broth containing Ampicillin (269 μM) or Triclosan (1, 5, or 10 μM). The seed culture was grown at 30°C while shaking at 250 rpm until OD600 reached to ~1. The seed cultures were centrifuged at 3000 x g for 10 min, re-suspend into fresh culture medium containing Ampicillin, and used to inoculate the main culture at a 1:20 dilution (~0.05 OD600) in a baffled flask. Re-suspension in fresh medium was not carried out for cultures containing Triclosan. The cultures were grown at 30°C while shaking at 250 rpm until OD600 reached to 0.5–0.6. The cultures were then equilibrated to induction temperature (37, 30, or 22°C) and expression was induced with 50 μM of Isopropylthio-β-galactoside (IPTG). Induced cultures were grown for different time lengths (4 h at 37°C, 6 h at 30°C or 12 h at 22°C) before pelleting bacterial cells by centrifuging at 5000 x g for 10 min in pre-weighed centrifuge tubes/ bottles.

### Cell lysis, protein extraction and purification, and SDS-PAGE/western blot analyses

Cell lysis, protein extraction, and purification were performed essentially as described previously [20, 21]. Briefly, bacterial cells were lysed with B-Per extraction reagent (Thermo Scientific, #78248) supplemented with DNAse I (Thermo Scientific, #90083) and protease inhibitor cocktail (Thermo Scientific, #87785) following manufacturer’s instructions. Clear supernatant containing soluble proteins was passed through 0.45 μm membrane (Millipore, #HPWP04700) and purified using HisPur Cobalt resin (Thermo Scientific, #89965) following supplier’s protocols. Purified proteins were dialyzed against PBS using Slide-A-Lyzer Dialysis Cassettes, 10K MWCO (Thermo Scientific, #66710) and stored at -80°C in small aliquots. SDS-PAGE and western blot analyses were performed following standard protocols and as described previously [20]. Intensities of immunoreactive bands on western blots were quantified by densitometric analysis performed with ImageJ software [22].

### Plasmid retention/stability assay

Plasmid retention assay was performed as described previously [23] with modifications. BL21(DE3) *E*. *coli* were transformed with expression vectors. A single colony from selection plate was used to inoculate 2 mL LB broth containing 269 μM Ampicillin or 1 μM Triclosan and grown while shaking at 250 rpm for 18 h at 30°C. Next day, culture was diluted to 0.05 OD600 in fresh LB broth containing 269 μM Ampicillin or 1 μM Triclosan and continued to grow until OD600 reached to 0.5. At this point, culture was split into two fractions; one was left uninduced whereas the other was induced with 50 μM IPTG. Both induced and uninduced cultures were grown under different culture condition i.e. 4 h at 37°C, 6 h at 30°C, or 12 h at 22°C. Cultures were then serially diluted and 250 μL of 10^−5^ and 10^−6^ dilutions were plated on non-selective LB agar and incubated for 18 h at 30°C. Next day, 100 colonies were patched on LB agar plates with or without selection markers. These plates were incubated for 18 h at 30°C, after which number of colonies were counted, and results reported as the percentage of cells retaining the plasmid vectors. These experiments were done in duplicate and on three different occasions.

### Antibiotic compatibility assay

BL21(DE3) *E*. *coli* were transformed with expression vectors and selected on LB agar plates containing 269 μM Ampicillin or 1 μM Triclosan. From selection plate, a single colony was used to inoculate 10 mL of LB broth containing 269 μM Ampicillin or 1 μM Triclosan and cultures were grown for 18 h at 30°C. The seed culture was then diluted with fresh LB broth to 0.1 OD600 and spread on agar plates containing 269 μM Ampicillin or 1 μM Triclosan. The agar plates were divided into three equal quadrants and antibiotic assay discs (GE Healthcare Life Sciences, #2017–006) impregnated with Chloramphenicol (25 μg), Kanamycin (50 μg), or Tetracycline (10 μg) were placed. The agar plates were incubated for 18 h at 30°C and zones of inhibition (ZOI) were measured.

### Statistical analysis

Statistical analyses were performed using SPSS software. Statistical analysis was carried out using the independent t-test to determine the significant differences between mean values of the groups. Within a group, one-way ANOVA was used to compare between samples of the group. For further analysis, a two-way ANOVA was used in determining the interaction between two factors/groups for this study. Descriptive analysis was used in presenting the mean and standard deviation of the variables studied. The significance value was set at less than 0.05 (p<0.05).

## Results

### Comparison of recombinant protein expression between vectors containing *bla* or *fabV* as selection markers

**Fig 1:**
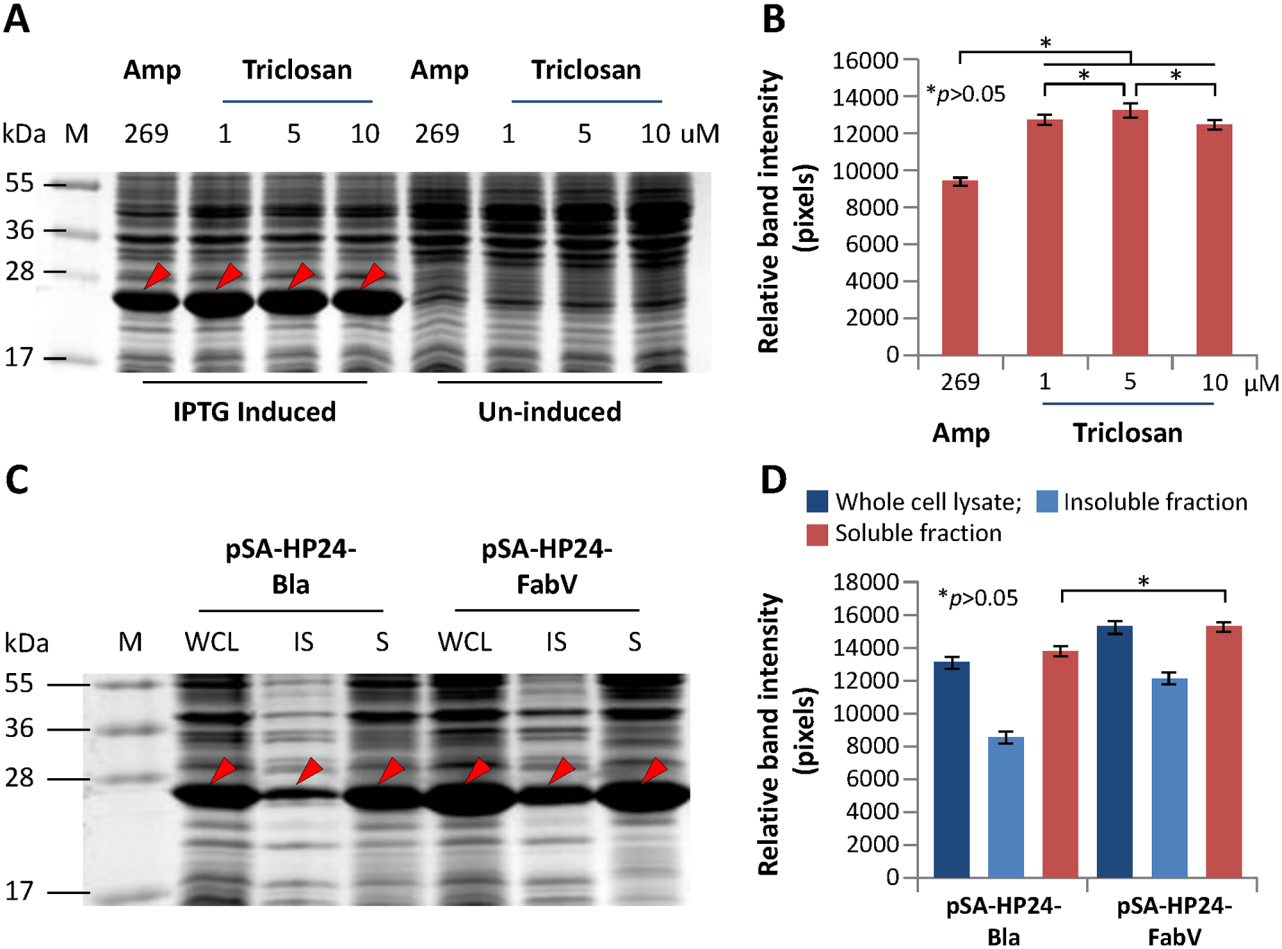
Comparison of recombinant HIV-1 P24 expression between vectors containing *bla* or *fabV* as selection markers. BL21(DE3) cells were transformed with pSA-HP24-Bla or pSA-HP24-FabV plasmids and cultured in LB broth supplemented with either 269 μM (100 μg mL^-1^) Ampicillin or 1, 5, 10 μM Triclosan respectively. Protein expression was induced with 50 μM IPTG when cultures reached to 0.5 OD600. Induced and uninduced cultures were grown at 30°C for 6 hours and then analysed by SDS-PAGE and densitometry for the expression of recombinant P24 protein (A & B). For further analysis, bacteria were lysed and proteins partitioned between insoluble and soluble fraction that were then analysed by SDS-PAGE and densitometry (C & D). Error bars show standard deviations calculated from at-least three independent experiments each performed in duplicate.

We used HIV-1 P24 as a model protein to compare recombinant protein expression between vectors containing *bla* or *fabV* selection markers. To do that, we transformed BL21(DE3) *E*. *coli* with pSA-HP24-Bla or pSA-HP24-FabV vectors and grew the cultures in presence of either Ampicillin or Triclosan. Upon IPTG induction, P24 expression from Amp or FabV vectors was statistically indifferent. (Fig 1A and 1B). To find out optimal concentration of Triclosan for recombinant protein expression, bacteria transformed with FabV vector were individually cultured in LB broth containing 1, 5, or 10 μM Triclosan. However, there was no difference in P24 expression between the cultures that were grown in presence of different Triclosan concentrations (Fig 1A and 1B). These data suggested that 1 μM Triclosan was sufficient for optimal recombinant protein expression from FabV vectors. After cell lysis, the presence of P24 in the soluble and insoluble fractions was monitored by SDS-PAGE and western blot of the samples. Solubility of recombinant P24 remained unchanged between bacterial cultures expressing P24 from Bla or FabV vectors (Fig 1C and 1D). Although cultures expressing P24 from FabV vector appeared to give better recombinant protein yields, these differences were statistically insignificant (Fig 1D).

### FabV/Triclosan selection system is compatible with culture media commonly used for recombinant protein expression

**Fig 2:**
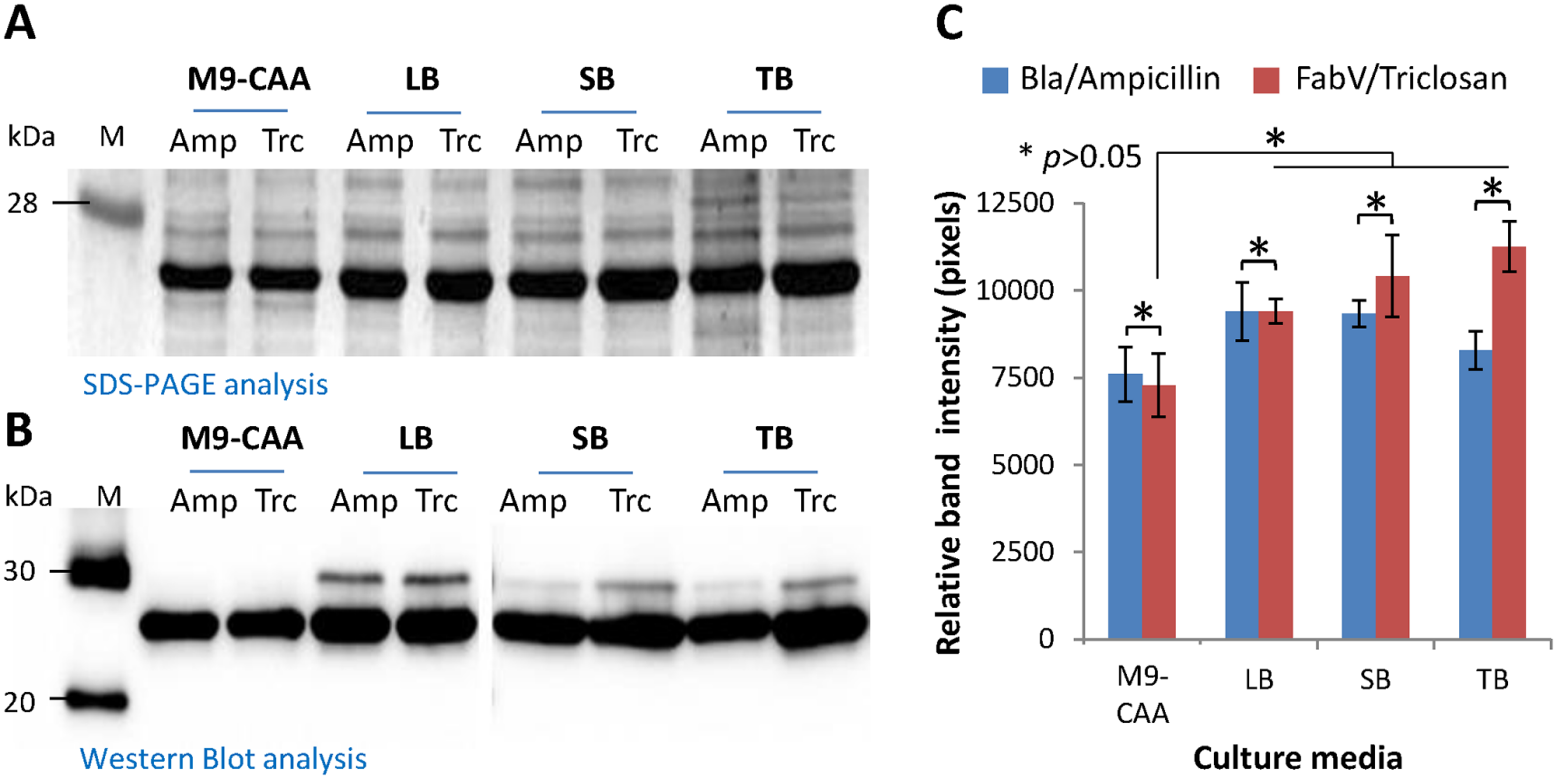
FabV-Triclosan selection system is compatible with culture media commonly used for recombinant protein expression. BL21(DE3) cells were transformed with pSA-HP24-Bla or pSA-HP24-FabV plasmids and cultured in different culture broths supplemented with either 269 μM (100 μg mL^-1^) Ampicillin or 1 μM Triclosan respectively. Protein expression was induced with 50 μM IPTG when cultures reached to 0.5 OD600. Induced and uninduced cultures were grown at 30°C for 6 hours and then analyzed by SDS-PAGE (A), Western blot (B), and densitometry (C) for the expression of recombinant P24 protein. Error bars show standard deviations calculated from at-least three independent experiments each performed in duplicate.

BL21(DE3) *E*. *coli* were transformed with pSA-HP24-Bla or pSA-HP24-FabV vectors and seeded into four commonly used culture media *i*.*e*. M9-CAA, LB, SB, and TB containing either Ampicillin or Triclosan. Cultures were grown until OD600 reached 0.5. IPTG was added to induce recombinant protein expression and cultures were grown at 30°C for 6 h. Total proteins were extracted from normalized bacterial biomass and subjected to SDS-PAGE and western blot analyses. Expression of recombinant P24 from FabV and Bla vectors was comparable in all four culture media (Fig 2A and 2B). Expression of recombinant P24 appeared to be low in M9-CAA medium when compared with rich media but this difference was statistically insignificant (Fig 2C). These data show that FabV-Triclosan selection system is compatible with culture media normally used for recombinant protein expression.

### Culture growth, protein expression kinetics, and recombinant protein yields

**Fig 3:**
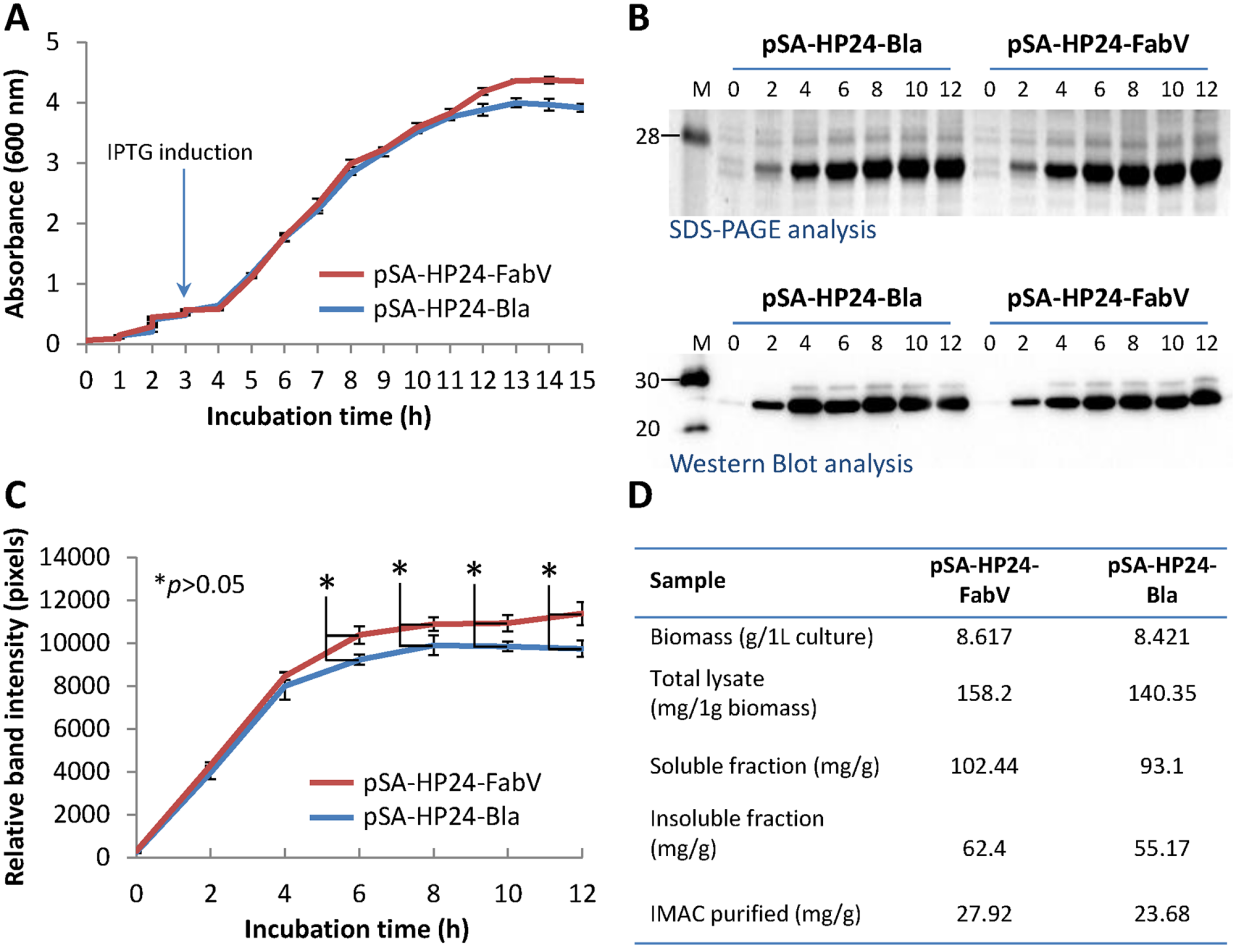
Culture growth, protein expression kinetics, and recombinant protein yields. BL21(DE3) cells were transformed with pSA-HP24-Bla or pSA-HP24-FabV plasmids and cultured in LB broth supplemented with either 269 μM (100 μg mL^-1^) Ampicillin or 1 μM Triclosan respectively. Protein expression was induced with 50 μM IPTG when cultures reached to 0.5 OD600. Induced and uninduced cultures were grown at 30°C for 15 h. Culture samples were collected at 1 h interval and used to monitor bacterial growth (A). Samples collected at various times points were analysed for recombinant P24 production (B) and protein expression kinetics (C). Recombinant P24 was purified from and biomass and protein yield compared between two cultures (D). Error bars show standard deviations calculated from at-least three independent experiments each performed in duplicate.

Next we compared the growth rate, protein expression kinetics, and recombinant protein yields between the cultures expressing P24 from Bla or FabV vectors. Cultures were grown at 30°C and sampled every 1 h for up to 15 h. We did not observe significant differences in growth kinetics of BL21(DE3) expressing recombinant P24 from Bla or FabV plasmids (Fig 3A). Samples harvested at regular intervals (2 h) revealed a steady increase in protein expression that peaked at 6–8 h post induction. Protein expression kinetics was indifferent between cultures expressing recombinant P24 protein from either Bla or FabV plasmids (Fig 3B and 3C). Cultures were upscaled to 1 L and used to purify recombinant P24 to homogeneity. Total biomass, protein present in total cell lysates, insoluble, and soluble fractions, and final recombinant P24 yields were similar between the cultures expressing P24 from Bla and FabV vectors (Fig 3D). These results showed that expression and production of recombinant P24 was similar between Bla-Ampicillin and FabV-Triclosan selection systems.

### Expression of other heterologous recombinant proteins

**Fig 4:**
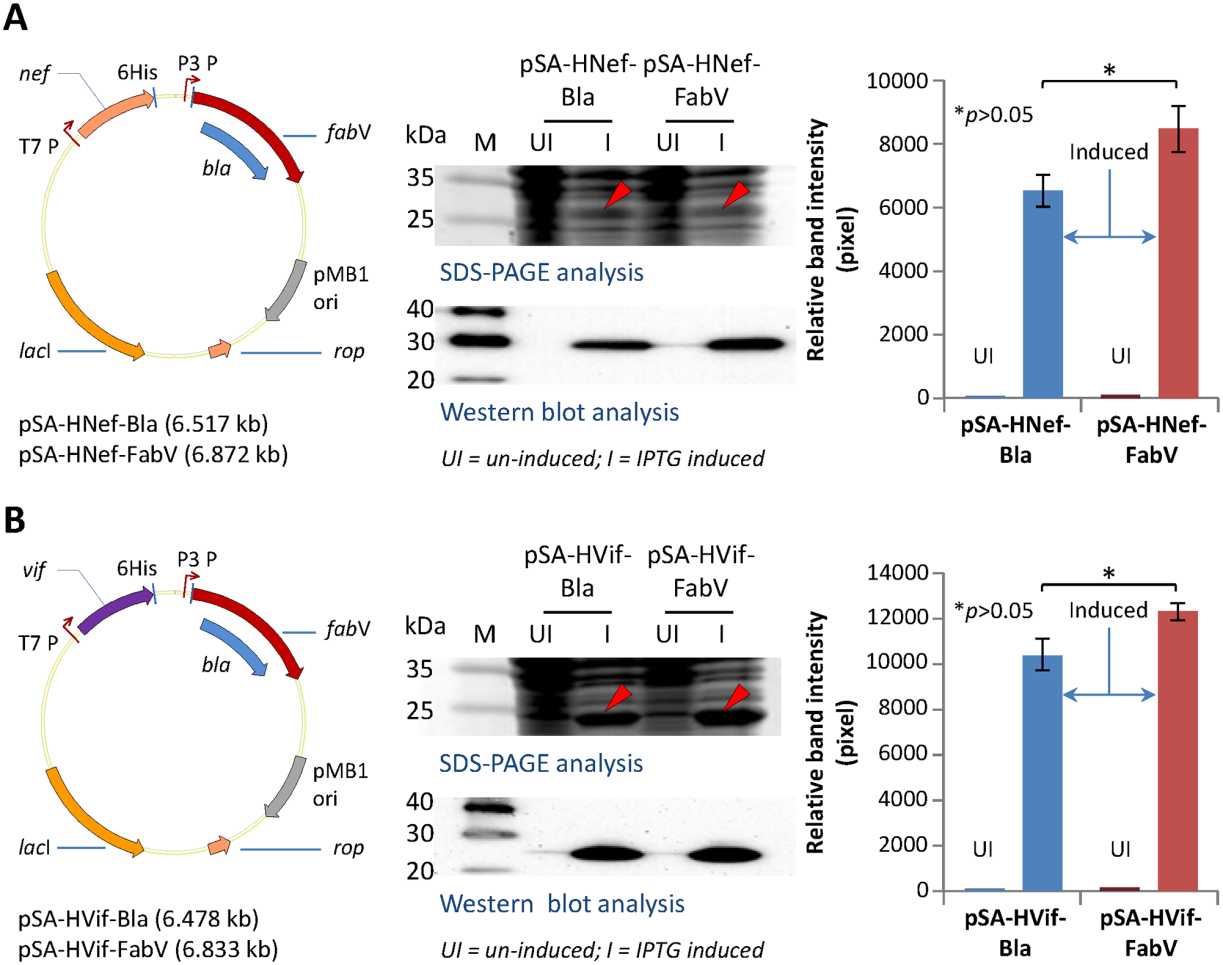
Expression of other heterologous recombinant proteins. BL21(DE3) cells were transformed with pSA-Nef/Vif-Bla or pSA-Nef/Vif-FabV plasmids and cultured in LB broth supplemented with either 269 μM (100 μg mL^-1^) Ampicillin or 1 μM Triclosan respectively. Protein expression was induced with 50 μM IPTG when cultures reached to 0.5 OD600. Induced and uninduced cultures were grown at 30°C for 6 hours and then analyzed by SDS-PAGE, western blot, and densitometry for the expression of recombinant Nef (A) and Vif (B) proteins. Error bars show standard deviations calculated from at-least three independent experiments each performed in duplicate.

To further validate the utility of FabV-Triclosan selection system, we expressed another two proteins, namely HIV-1 Nef and HIV-Vif. Vectors pSA-HNef-Bla and pSA-HVif-Bla [21] were modified by replacing *bla* gene with *fabV* gene as described in material and methods section. BL21(DE3) were then transformed with pSA-HNef-Bla, pSA-HNef-FabV, pSA-HVif, or pSA-HVif-FabV vectors and used to express Nef and Vif proteins. Expression of both Nef and Vif proteins from FabV vectors was comparable with expression of these proteins from Bla vectors (Fig 4A and 4B). These data confirmed that FabV-Triclosan can be used to express an assortment of heterologous recombinant proteins in *E*. *coli*.

### Plasmid retention/stability and protein expression

Loss of expression vector from host bacteria may result in reduced production of recombinant heterologous protein [19, 23]. To find out if FabV vectors were more stable than Bla vectors, we transformed BL21(DE3) *E*. *coli* with Bla or FabV expression vectors. Colonies from freshly streaked plates were used to inoculate seed cultures that were then diluted with fresh media containing either Ampicillin or Triclosan. Cultures were either induced with IPTG or left uninduced and grown at 37, 30, and 22°C for 4, 6, and 12 h respectively. Cultures were appropriately diluted and plated on agar plates and plasmid retention was assayed by patching colonies on agar plates with or without selection markers.

**Fig 5:**
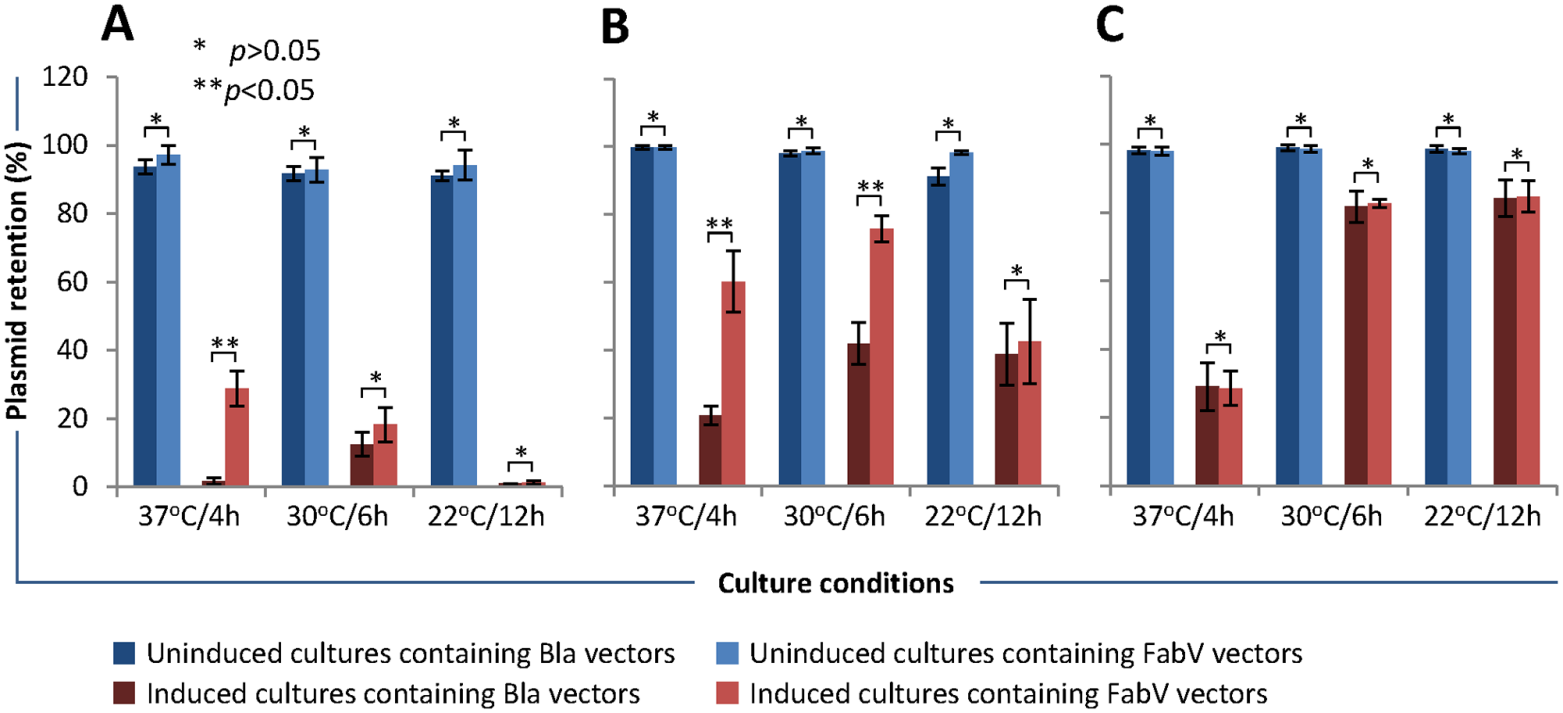
Comparison of plasmid stability/retention between Bla and FabV vectors. BL21(DE3) cells were transformed with Bla or FabV vectors and uninduced and IPTG -induced cultures were grown at 37°C/4h, 30°C/6h, and 22°C/12 h in presence of Ampicillin (for bacteria containing Bla vectors) or Triclosan (for bacteria containing FabV vectors). Cultures were diluted and plated on non-selective LB agar plates. After 18 h incubation at 30°C, 100 colonies were replica patched on non-selective and selective (with Ampicillin or Triclosan) LB agar plates, incubated at 30°C for 18 h, and counted. (A) cultures expressing P24 protein; (B) cultures expressing Nef protein; (C) cultures expressing Vif protein. Error bars show standard deviations calculated from three independent experiments each performed in duplicate.

FabV vectors expressing, recombinant P24 protein were more stable than Bla vectors in cultures grown at 37°C/4 h (28.75% vs 1.75%) and 30°C/6 h (18.25% vs 12.5%). In cultures grown at 22°C/12 h, plasmid retention was similar between FabV and Bla vectors (1.25% vs 1.00%). More than 90% bacteria retained plasmids in un-induced cultures under all three culture conditions (Fig 5A). Similarly, FabV vectors expressing recombinant Nef protein were more stable than Bla vectors in cultures grown at 37°C/4 h (60.25% vs 20.75%) and 30°C/6 h (75.75% vs 42%). At 22°C/12 h, both FabV and Bla vectors were retained similarly (42.50% vs 38.75%). In un-induced cultures, between 91–99% bacteria retained the plasmids under all three culture conditions (Fig 5B). Interestingly, FabV and Bla vectors expressing Vif protein were retained similarly under all three culture conditions i.e. 37°C/4 h (28.75% vs 29.00%), 30°C/6 h (82.75% vs 81.75%), and 22°C/12 h (84.75% and 84.25%). In uninduced cultures, 98–99% bacteria retained plasmids under all three culture conditions (Fig 5C). These data showed that selection marker (FabV or Bla) alone was not determining the plasmid stability but the recombinant protein being expressed in bacterial host was also contributing towards plasmid stability. Nonetheless, for at-least two proteins (P24 and Nef), FabV vectors were clearly more stably retained by the host *E*. *coli*.

**Fig 6:**
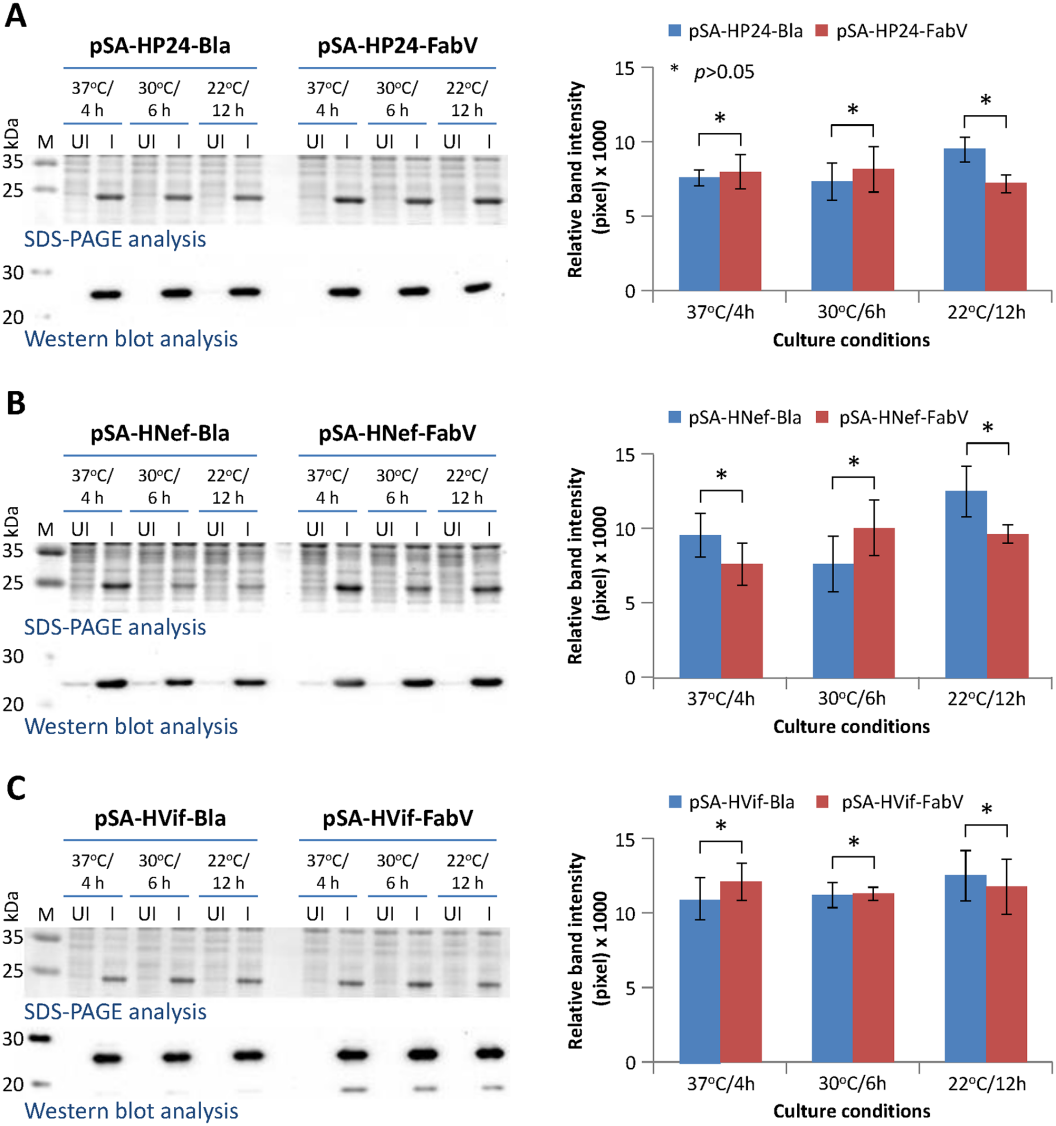
Effect of plasmid retention/stability on protein expression. BL21(DE3) cells were transformed with Bla or FabV vectors and uninduced and IPTG -induced cultures were grown at 37°C/4h, 30°C/6h, and 22°C/12 h in presence of Ampicillin (for bacteria containing Bla vectors) or Triclosan (for bacteria containing FabV vectors). (A) Cultures expressing P24 protein; (B) cultures expressing Nef protein; (C) cultures expressing Vif protein. Left panel shows SDS-PAGE/western blots and right panel shows densitometric analyses of bands on western blots. Error bars show standard deviations calculated from three independent experiments each performed in duplicate.

Next we determined if plasmid retention would affect the expression of recombinant heterologous proteins. Cultures were normalized, lysed, and extracted proteins subjected to SDS-PAGE and western blot analyses. Plasmid retention did not appear to correlate with recombinant protein expression. Recombinant P24, Nef, and Vif expression from FabV and Bla plasmids was similar under all three culture conditions tested (Fig 6A, 6B, and 6C).

### FabV-Triclosan selection system is compatible with other antibiotics

**Fig 7:**
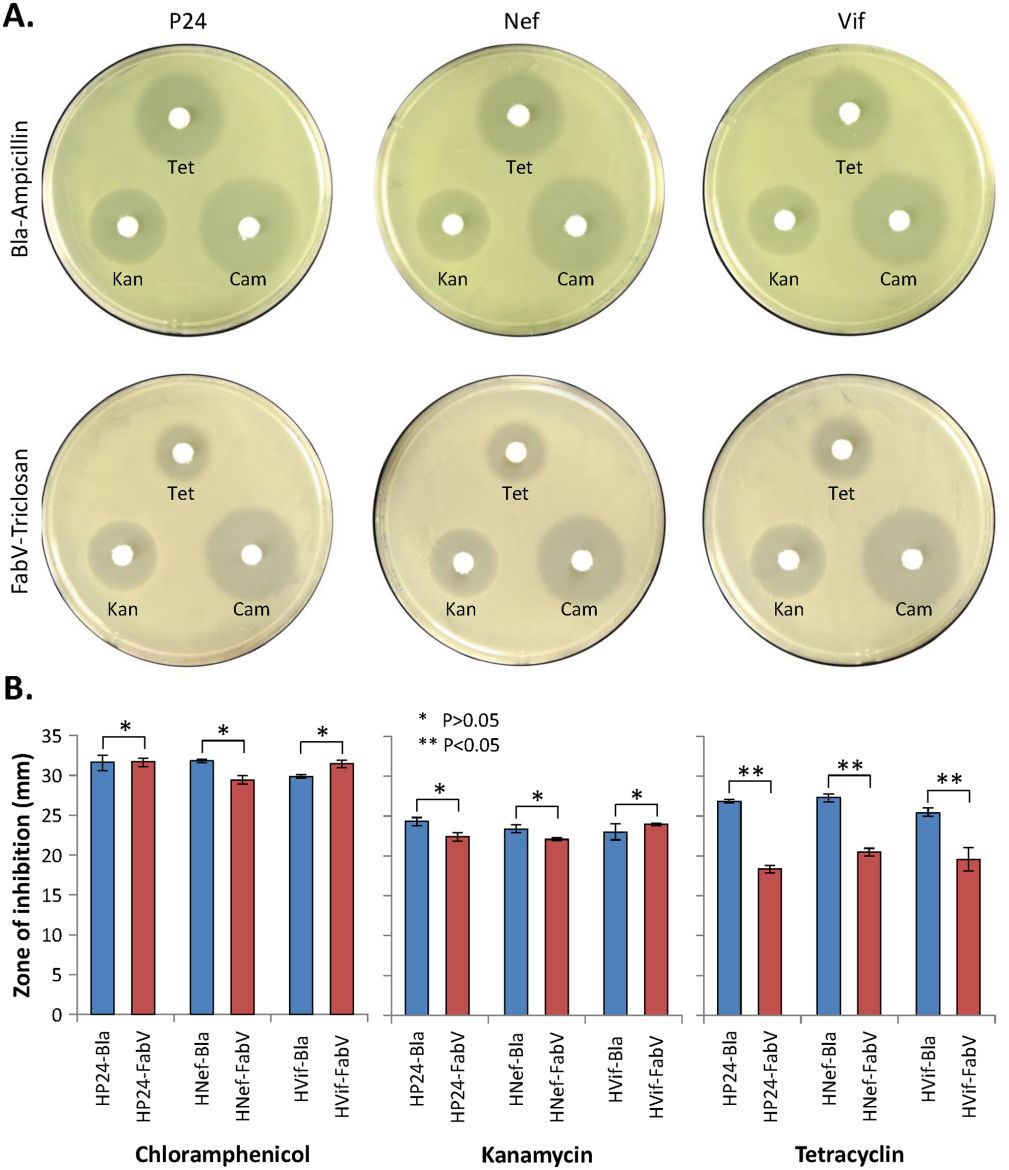
FabV-Triclosan selection system is compatible with other antibiotics. BL21(DE3) harboring Bla or FabV vectors were spread on agar plates containing either Ampicillin or Triclosan. Antibiotic sensitivity discs impregnated with Chloramphenicol (25 μg), Kanamycin (50 μg), or Tetracycline (10 μg) were placed. Following overnight incubation, zones of inhibition were measured and compared between the two selection systems. Error bars show standard deviations calculated from at-least three independent experiments each performed in duplicate.

Bacterial host may harbor helper plasmids that assist with tighter control of expression, supply chaperons for proper protein folding, or express rare tRNAs. These helper plasmids usually contain *cat*, *aphA*, or *tetA* genes and maintained in presence of Chloramphenicol, Kanamycin, or Tetracyclin respectively. We were therefore interested to know if overexpression of FabV and presence of Triclosan in culture medium would affect the susceptibility of BL21(DE3) towards above-mentioned antibiotics. To find out, we spread BL21(DE3) harboring Bla or FabV expression vectors on agar plates containing either Ampicillin or Triclosan and placed antibiotic assay discs impregnated with Chloramphenicol, Kanamycin, or Tetracycline. Following overnight incubation, zones of inhibition were measured and compared between two selection systems. BL21(DE3) bacteria transformed with FabV vectors were just as susceptible to Chloramphenicol and Kanamycin as were the bacteria containing Bla vectors (Fig 7A). However, bacteria containing FabV vectors were significantly more resistant towards Tetracycline compared to bacteria containing Bla vectors as determined by reduced zone of inhibition. Expression of different proteins had no effect on zones of inhibition (Fig 7B). These data showed that FabV-Triclosan selection system can be used in combination with Chloramphenicol and Kanamycin but perhaps more Tetracycline would be needed for efficient maintenance of Tetracycline selectable helper plasmids.

## Discussion

This report is an extension of our previous work where we have shown that overexpression of *V*. *cholerae fabV* gene can be used to select pBR322 -based medium copy plasmid vectors in culture medium supplemented with Triclosan [18]. Since overexpression of heterologous proteins from plasmid vectors can be toxic and exert metabolic burden, heterologous genes are often expressed from medium-copy number expression vectors. A majority of these vectors harbor antibiotic resistance markers that help to maintain plasmid vectors inside the host cells when they are grown in presence of antibiotics. β-lactamase-Ampicillin is the most commonly used plasmid selection system. Unfortunately, β-lactamase is produced and secreted in ample amounts into the culture medium where it degrades Ampicillin. Furthermore, Ampicillin is susceptible to degradation by low pH and elevated temperatures during prolonged incubations. Consequently, bacteria that lack plasmid will not be eliminated and will begin to overgrow the culture that will eventually result in reduced recombinant protein expression. To alleviate these issues, bacteria are spun down to remove spent medium containing β-lactamase and inoculated in fresh medium supplemented with 100–200 μg mL^-1^ Ampicillin. Alternatively culture medium can be supplemented with more stable but expensive Carbenicillin.

Utilizing expression vectors engineered here and in the previous study [18], we demonstrate that FabV-Triclosan is an efficient alternative to traditional and commonly used Bla-Ampicillin selection system for the maintenance of expression vectors in *E*. *coli*. As a model protein, we expressed HIV-1 P24 from Bla and FabV plasmids in medium containing either Ampicillin or Triclosan. Expression of recombinant P24 from Bla and FabV vectors was similar. Compared to 269–538 μM (100–200 μg mL^-1^) Ampicillin that is necessary to maintain Bla plasmids for efficient recombinant protein expression, FabV plasmids can be retained in presence of just 1 μM Triclosan.

To test whether FabV-Triclosan selection could function well in different growth media, we grew bacteria containing FabV or Bla in four commonly used culture media and compared expression of recombinant P24 protein. Comparable recombinant P24 expression from both FabV and Bla vectors in all four culture media suggested that FabV-Triclosan enables efficient selection of plasmid vectors in commonly used bacterial media. In upscaled 1L cultures, final biomass, total expressed protein, and net yield of purified P24 was similar between FabV and Bla selection systems. To further validate the usefulness of FabV-Triclosan selection system, we compared the expression of Nef and Vif proteins from FabV and Bla vectors. Similar expression of Nef and Vif proteins from FabV and Bla vectors confirmed that FabV-Triclosan system can be used to express an assortment of recombinant heterologous proteins.

Goh and Good found FabI-containing pUC -based vectors were more stable than parental plasmids containing Bla as a selectable marker [15]. In agreement with their findings, we found FabV plasmids comparably more stable than Bla vectors but only at elevated temperatures (37 and 30°C) and shorter incubation times (4 and 6 h). At 22°C and after 12 h incubation, plasmid loss from IPTG -induced bacteria was similar between the two selection systems. Interestingly, compared to P24 and Nef, when Vif was expresses from Bla or FabV plasmids, FabV-Triclosan resulted in no better plasmid stability under all three culture conditions. These data suggest that plasmids stability was also dependent on heterologous protein being expressed. Based on these observations, we were expecting higher protein yields for P24 and Nef proteins when expressed from FabV vectors and at 37 and 30°C for 4 and 6 h respectively. Unexpectedly, we did not observe any better yields of P24 and Nef proteins and expression of these proteins appeared to be independent of number of bacteria that retained the expression vectors.

Certain bacterial host such as BL21(DE3)-pLysS (Novagen), BL21-AI (Invitrogen), Origami (Novagen) etc., are grown in presence of antibiotics such as Chloramphenicol, Kanamycin, or Tetracycline either for verification purposes or to maintain additional extra-chromosomal genetic elements such as pLysS vectors. Similarly, helper plasmids expressing chaperons or genes for rare tRNAs or genes assisting with disulfide bond formation (*dsbA*, *dsbC*) etc., are sometimes needed for efficient expression of certain proteins. We anticipated that there will be instances where FabV vectors will be maintained together with vectors containing other antibiotic selection markers. It was therefore important to learn if FabV-Triclosan system was compatible with other antibiotics. We found that FabV -transformed and Triclosan selectable cultures were as sensitive to Chloramphenicol and Kanamycin as were those that contained Bla vector and selected on Ampicillin. However, *E*. *coli* containing FabV vector were significantly more resistant towards Tetracycline. Resistance to Tetracycline is achieved through three major mechanisms i.e. Tetracycline efflux, ribosome protection, and Tetracycline modification [24]. How overexpression of FabV affects these mechanisms needs further investigation. These findings however suggest that FabV-Triclosan is compatible with other antibiotics and overexpression of FabV resulted in enhanced but not total resistance towards Tetracycline.

In conclusion, this study demonstrates that: **a)** FabV-Triclosan plasmid selection system can be used efficiently to express heterologous recombinant proteins; **b)** FabV vectors are relatively more stable than Bla vectors; **c)** FabV-Triclosan selection system works well in various culture media; **d)** FabV-Triclosan selection system is compatible with other selection markers used to maintain plasmid vectors. In addition to above mentioned features, the real added value of our proposed system is the utilization of a biocide agent and at a fraction of the amount (1 μM Triclosan versus 269μM Ampicillin) of an antibiotic that makes this system environmental friendly.
